# Role of cell-free DNA levels in the diagnosis and prognosis of sepsis and bacteremia: A systematic review and meta-analysis

**DOI:** 10.1371/journal.pone.0305895

**Published:** 2024-08-29

**Authors:** Mohammad Najm Dadam, Le Thanh Hien, Engy M. Makram, Lam Vinh Sieu, Ahmad Morad, Nada Khalil, Linh Tran, Abdelrahman M. Makram, Nguyen Tien Huy

**Affiliations:** 1 Department of Geriatrics, Helios Clinic Schwelm, Schwelm, Germany; 2 Online Research Club, Nagasaki, Japan; 3 Department of Obstetrics and Gynecology, Ho Chi Minh City Medicine and Pharmacy University, Ho Chi Minh City, Vietnam; 4 College of Medicine, Misr University for Science and Technology, Giza, Egypt; 5 Faculty of Medicine, Moscow State University of Medicine and Dentistry Named After A.I. Yevdokimov, Moscow, Russia; 6 Faculty of Medicine, Ain Shams University, Cairo, Egypt; 7 School of Medicine, New Giza University, Giza, Egypt; 8 School of Medicine, Vietnam National University Ho Chi Minh City, Ho Chi Minh City, Vietnam; 9 Vietnam National University Ho Chi Minh City, Ho Chi Minh City, Vietnam; 10 School of Public Health, Imperial College London, London, United Kingdom; 11 Institute of Research and Development, Duy Tan University, Da Nang, Vietnam; 12 School of Medicine and Pharmacy, Duy Tan University, Da Nang, Vietnam; 13 School of Tropical Medicine and Global Health, Nagasaki University, Nagasaki, Japan; Weill Cornell Medicine, UNITED STATES

## Abstract

**Background:**

Sepsis remains a major cause of mortality in intensive care units (ICUs). Prompt diagnosis and effective management are imperative for better outcomes. In this systematic review and meta-analysis, we explore the potential of circulating cell-free DNA (cfDNA), as a promising tool for early sepsis detection and prognosis assessment, aiming to address limitations associated with traditional diagnostic methods.

**Methods:**

Following PRISMA guidelines, we collected relevant literature from thirteen databases. Studies were included if they analyzed quantitative diagnostic or prognostic cfDNA levels in humans in case of sepsis. We collected data on basic study characteristics, baseline patient demographics (e.g. age and sex), and cfDNA levels across different stages of sepsis. Pooled SMD with 95%-CI was calculated, and Comprehensive Meta-Analysis (CMA) software facilitated meta-analysis. Receiver operating characteristic (ROC) curves were generated to assess cfDNA’s combined sensitivity and specificity in diagnostics and prognostics.

**Results:**

We included a final of 44 studies, of which, only 32 with 2950 participants were included in the meta-analysis. cfDNA levels were higher in septic patients compared to healthy controls (SMD = 3.303; 95%-CI [2.461–4.145], p<0.01). Furthermore, cfDNA levels were higher in non-survivors than survivors (SMD = 1.554; 95%-CI [0.905–2.202], p<0.01). Prognostic studies demonstrated a pooled sensitivity and specificity of 0.78, while diagnostic studies showed a sensitivity of 0.81 and a specificity of 0.87.

**Conclusion:**

These findings show that cfDNA levels are significantly higher in sepsis patients compared to control groups and non-survivors in comparison to survivors among both adult and pediatric populations.

## Introduction

The bloodstream is the battlefield in the immune system’s ongoing fight against pathogens. When immune defenses weaken, it disrupts the balance, leading to complications. Bacteremia occurs when bacteria evade immune control and enter the circulation, defined as the presence of viable bacteria in the blood [1]. While usually temporary and cleared from the bloodstream, an overwhelmed immune system can result in a bloodstream infection, potentially escalating to sepsis [2].

Sepsis is defined as a critical impairment of organ function resulting from an imbalanced and dysfunctional response of the body to an infection, leading to a life-threatening condition, septic shock, or even death [3]. In 2017, the global incidence of sepsis reached an estimated 48.9 million cases, contributing to 11 million deaths. This accounted for approximately 19.7% of all global deaths in the same year [4], which is an indication of a rising trend over 20 years concerning hospitalizations [5–7] and overall mortality between 26% and 48% according to several multicenter cohort studies among intensive care unit (ICU) patients [8–10].

Because physicians face difficulties in the early diagnosis of sepsis [11], the diagnosis often relies on empirical assessments as late initiation of treatment can have deleterious effects [12]. While blood cultures confirm bacteremia, they often yield false negatives in 28–49% of sepsis cases [13, 14]. Additionally, the sensitivity and specificity of blood cultures are adversely affected by errors related to the volume and timing of blood collection and antibiotic therapy [15]. Recent research suggests that circulating cell-free DNA (cfDNA) may represent a more promising early diagnostic and prognostic biomarker for sepsis patients, akin to its established utility in the diagnosis of other conditions such as cancer [16–18].

cfDNA was first reported in 1948 by Mandel and Metais and has since been discovered in various bodily fluids [19, 20]. Current evidence suggests that cfDNA is released into the circulation from apoptotic and necrotic cells suggesting that cfDNA levels should physiologically be low [21, 22]. Numerous studies have demonstrated a notable distinction in cfDNA levels between patients who survive and those who do not in ICUs. A groundbreaking study by Martins et al. revealed the presence of circulating cfDNA in the plasma of septic patients [23]. This discovery marked the first evidence of cfDNA in sepsis, expanding possibilities for sepsis diagnosis and prognosis [24, 25].

Recent studies have shown that cfDNA can be 81% (95%-CI 75%-86%) sensitive and 72% (95%-CI 65%-78%) specific in identifying sepsis in critically ill patients [26]. Moreover, metagenomic sequencing can provide better prediction, where the area under the curve in one study reached 0.992 (95%-CI 0.969–1.000) for the diagnosis of sepsis [27]. Still, a meta-analysis for the diagnostic and prognostic applicability of cfDNA in bacteremia, sepsis, and septicemia is required.

In this systematic review and meta-analysis, we aimed to evaluate the use of cfDNA as a diagnostic and prognostic marker in patients suffering from sepsis in ICUs.

## Methods

### Protocol development and registration

This study followed the Preferred Reporting Items for Systematic Reviews and Meta-Analyses (PRISMA) Statement [28], as shown in S1 Table in S1 File. The study protocol was registered on the PROSPERO database on 28 April 2020 (CRD42020162215). For the conduct of this study, ethical approval and informed consent were not applicable as the data used are openly available without restrictions.

### Search strategy

On 22 September 2019, we conducted a systematic electronic search in thirteen databases: PubMed, Google Scholar, Scopus, ClinicalTrials, EMBASE, Virtual Health Library (VHL), WHO (ICTRP), Cochrane, Web of Science (WOS), metaRegister of Controlled Trials (mRCT), ScienceDirect, System for Information on Grey Literature in Europe (SIGLE), and WHO Library. Modification of search terms to accommodate each database is represented in S2 Table in S1 File. On May 16, 2023, we performed an updated search using the same methods. Relevant references of the included articles, as well as related or similar articles, were manually searched via PubMed and Google Scholar.

### Selection criteria

We included any original study that reports the role of cfDNA in the diagnosis and prognosis of sepsis, without restrictions on age, sex, race, ethnicity, language, country, or publication. Studies pertaining exclusively to mitochondrial cfDNA, bacterial DNA, the role of cfDNA in diseases other than sepsis-related pathology, and animal or in vitro studies were excluded. Moreover, studies that did not quantitatively measure cfDNA levels, non-original studies, abstracts, and literature not available in full text were also excluded. Non-English articles were initially translated using ChatGPT 3.5 and Google Translate, followed by a thorough review by a native speaker to ensure accuracy and fluency.

### Study selection

Three reviewers independently screened titles and abstracts obtained from the searches, evaluating them for eligibility based on our predetermined inclusion criteria. All titles and abstracts meeting the inclusion criteria were then retrieved in full text. Subsequently, three independent reviewers examined these full-text articles to determine eligibility. A senior reviewer was consulted for discussion to resolve any disagreements.

### Data extraction

A standardized sheet was formed by a senior author through screening the full text of included articles. Pilot extraction tasks were performed, and modifications to the sheet were done. The final extraction sheet contained the following headers: study design, sample size, age, gender, follow-up duration, cfDNA timing and method of measurement, sepsis scores, area under the curve (AUC), sensitivity, and specificity. Due to various definitions of sepsis in the included studies, we collected all sepsis definitions. The extraction was done by three independent reviewers and then discussed between them to reach a final consensus. When cfDNA measures were only presented graphically, we attempted to contact the authors to obtain the numerical data. In case of unresponsiveness, WebPlotDigitizer was used to extract the values from the graphs [29].

### Data analysis

In cases where multiple blood samples were collected during a patient’s stay, the first detected cfDNA value after admission was utilized for analysis. The mean level of cfDNA in addition to the sample size of each study group was utilized to estimate the standardized mean difference (SMD) between the compared groups. This approach enabled a more comprehensive assessment of the cfDNA levels across the studies. The mean values and standard deviations for the data, originally provided as medians and ranges or interquartile ranges (IQR), were estimated using the statistical formula described by Wan et al. [30]. Comprehensive Meta-Analysis (CMA) software (Version 3.3.07, Biostat, NJ, USA) was used to conduct meta-analysis. Heterogeneity across the studies was measured using I^2^ statistic and p-value. If the I^2^ was >50% or p-value < 0.1, heterogeneity was considered statistically significant, and a random effects model was used [31]. If the heterogeneity was statistically insignificant, a fixed effects model was used. Standardized mean difference (SMD) was represented with their 95% confidence intervals (CI) in forest plots. We assessed publication bias through the examination of a funnel plot and Egger’s test [32]. In the presence of publication bias, we planned to conduct the Duval and Tweedie nonparametric trim and fill analysis to address and adjust for this bias [33]. Qualitative data analysis was done narratively through the discussion of twelve studies in the discussion section.

### Sensitivity and specificity analysis

To perform Hierarchical Summary receiver operating characteristic (HSROC) analysis, we employed the MetaDTA web-based tool. This user-friendly tool facilitates the meta-analysis of diagnostic test accuracy (DTA) studies and enables the generation of HSROC curves [34, 35]. The HSROC curve effectively summarizes diagnostic test performance across multiple studies by incorporating hierarchical modeling to account for within-study and between-study variability [36].

The pooled sensitivity and specificity were estimated using the meta4diag package, an R package designed for Bayesian bivariate meta-analyses of diagnostic test studies [37]. The applicability of cfDNA as a diagnostic marker for sepsis was further evaluated using likelihood ratios. A positive likelihood ratio (LR+) exceeding 10 indicates strong support for the diagnostic utility of cfDNA in sepsis, while a negative likelihood ratio (LR-) below 0.1 suggests that cfDNA can effectively rule out sepsis [38].

### Quality assessment

We employed the QUADAS-2 tool to assess diagnostic accuracy study quality [39]. As for prognostic studies, we used the modified QUAPAS tool [40].

## Results

### Study characteristics

**Fig 1:**
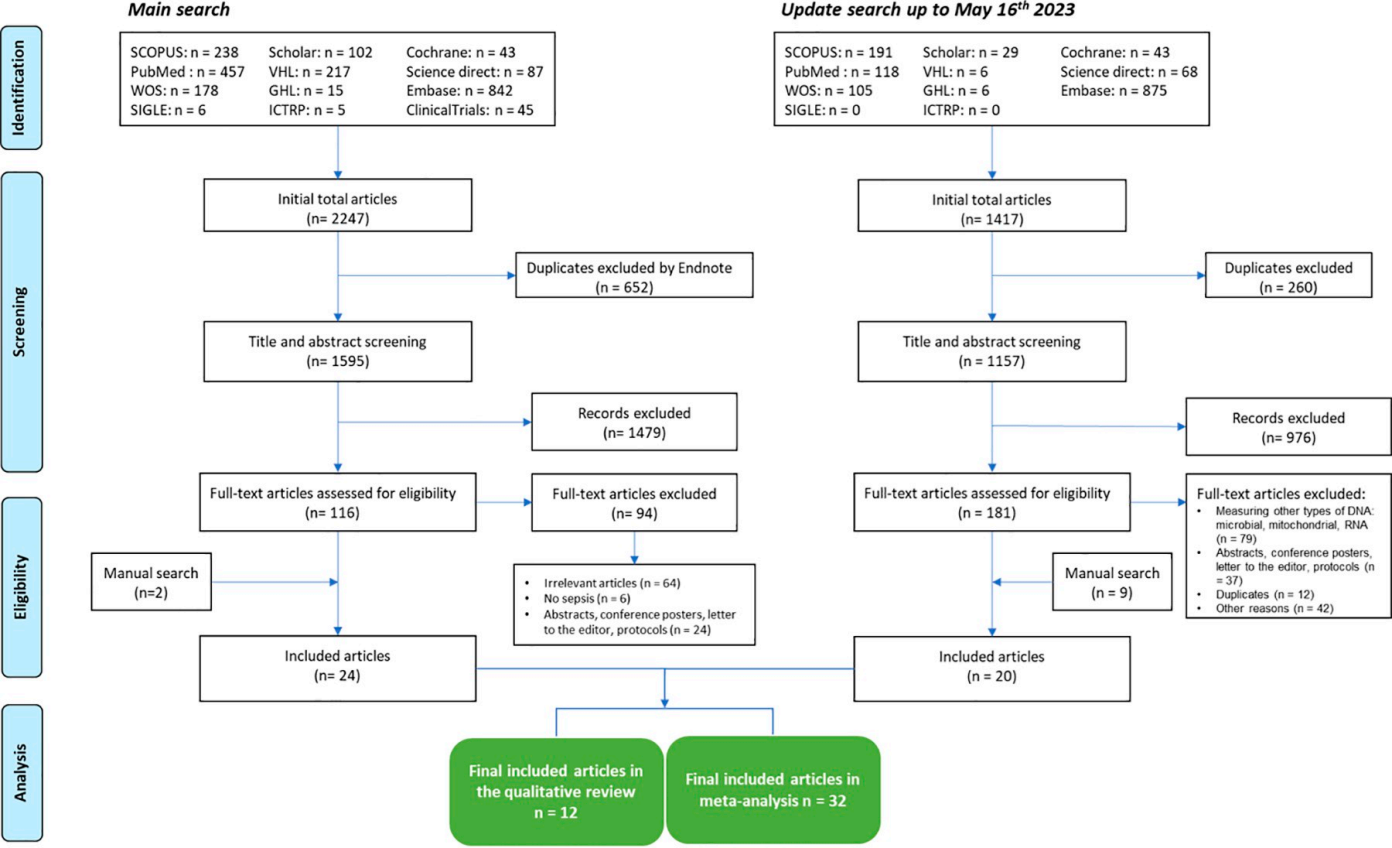
PRISMA flow diagram of studies’ screening and selection.

The initial search yielded 2247 eligible papers, of which 652 were excluded due to duplicity. Updated research gave 1417 articles, among them 260 were duplicates (Fig 1). A comprehensive review of 32 studies encompassing a total sample size of 2950 participants, both septic and non-septic, was conducted. Most septic patients were hospitalized in ICUs, prompting the collection of ICU scoring systems, such as SOFA and APACHE. Follow-up durations ranged from three days to one year. While most studies monitored participant mortality and survival outcomes, not all specifically focused on the prognostic potential of cfDNA. Detailed information regarding patient baseline characteristics, including age, follow-up duration, cfDNA measurement methods, and sepsis scores, is presented in S3 Table in S1 File. Moreover, the key features of the twelve qualitative articles are detailed in S4 Table in S1 File.

### Qualitative review

In addition to the 32 primary articles, we included 12 additional articles in our discussion. These articles addressed the same scope as our primary research, but data extraction was challenging, or they deviated slightly from our inclusion criteria. Out of these 12 articles, three focused specifically on patients with bacteremia and explored the diagnostic and prognostic implications of cfDNA in this population [41–43].

### Quantitative analysis

#### Circulating cfDNA levels in septic patients and control groups

A total of 25 studies [25, 27, 44–66] were included in the final comparison of the mean levels of cfDNA in adult patients with sepsis (n = 930) to the mean levels of cfDNA in non-septic patients (n = 690) (S1 Fig in S1 File). A combined result showed that the levels of cfDNA were significantly higher in septic patients when compared to healthy controls (SMD = 3.303; 95%-CI [2.461–4.145], p<0.01), ICU non-septic patients (SMD = 1.577; 95%-CI [2.142–4.557], p<0.01), and postoperative patients (SMD = 1.241; 95%-CI [2.142–4.557], p<0.01). Elevated levels of cfDNA have also been observed in pediatric sepsis patients [67–69]. Septic infants or children exhibited significantly higher cfDNA levels compared to controls (SMD = 1.584; 95%-CI [0.418–2.750], p = 0.008) (S1 Fig in S1 File).

#### Circulating cfDNA levels in deceased and surviving patients

**Fig 2:**
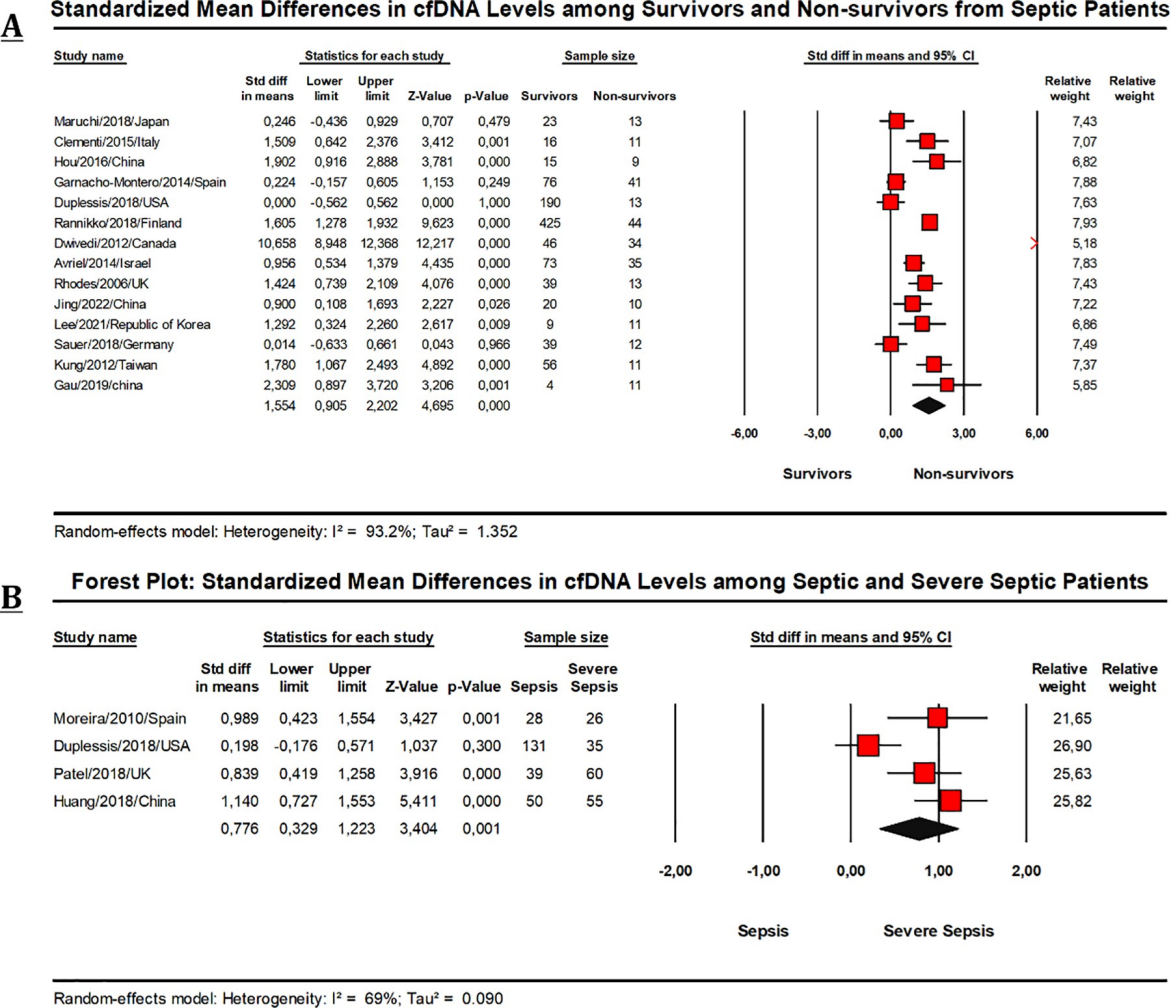
Forest plots for comparison of standardized mean difference (SMD) for prognostic purposes.

A total of 14 studies [25, 27, 45, 48, 51, 54–56, 62, 63, 70–73] were included in the final comparison of the levels of cfDNA in sepsis survivors (n = 1031) to the levels of cfDNA in sepsis non-survivors (n = 268) (Fig 2). The pooled SMD indicated that cfDNA levels were substantially higher in sepsis non-survivors compared to survivors (SMD = 1.554; 95%-CI [0.905–2.202], p<0.01), suggesting that cfDNA levels may be helpful biomarker for forecasting the likelihood of death in sepsis patients.

#### Differentiating severe and non-severe sepsis using cfDNA levels

A meta-analysis of four studies found that patients with severe sepsis or septic shock had significantly higher cfDNA levels than patients with a normal course of sepsis [52, 58, 60, 71] (Fig 2). The pooled SMD was 0.776 (95%-CI [0.329–1.223], p = 0.001), showing the potential for distinguishing between severe and non-severe sepsis.

#### Prognostic value of cfDNA levels in predicting outcomes in bacteremia patients

In a meta-analysis of two studies comparing cfDNA levels between bacteremia survivors and non-survivors at one month, we found significantly higher cfDNA levels in non-survivors (pooled SMD = 0.996, 95% CI [0.737, 1.256], p < 0.001). These findings suggest cfDNA’s potential as a prognostic marker for the infection course in bacteremia patients [41, 42].

#### Diagnostic and prognostic utility of cfDNA—Pooled sensitivity and specificity insights

**Fig 3:**
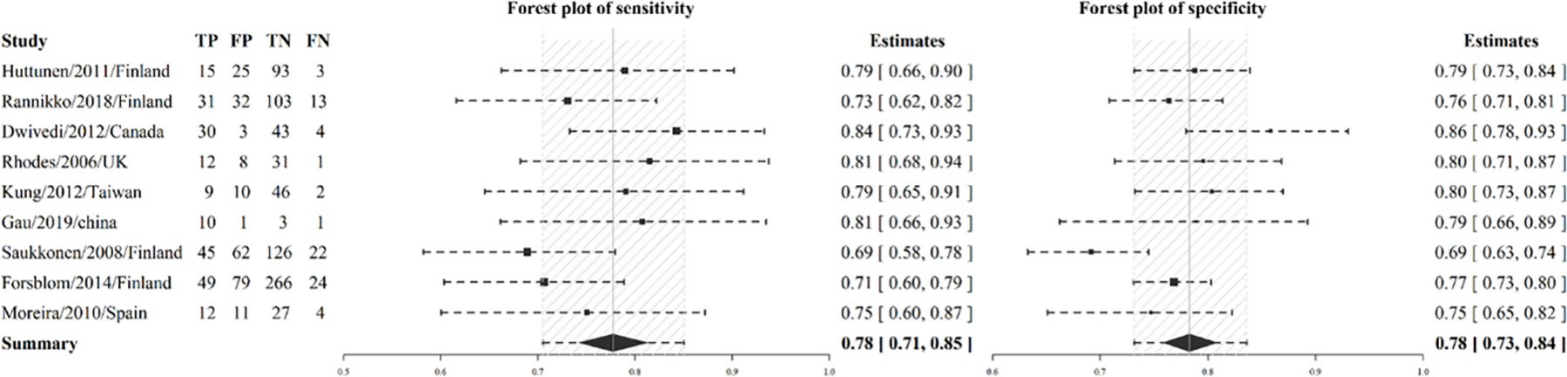
Forest plot of pooled sensitivity and specificity for prediction mortality among patients.

**Fig 4:**
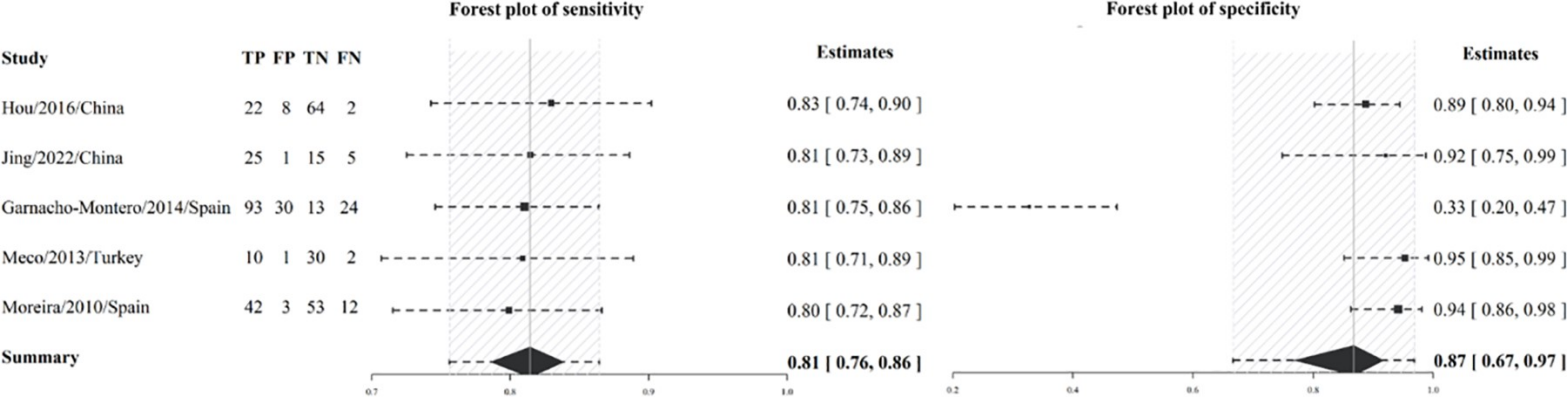
Forest plot of pooled sensitivity and specificity for sepsis diagnosis using cfDNA.

In this review, we incorporated nine prognostic and five diagnostic studies into our analysis to assess sensitivity and specificity. For a comprehensive overview of the prognostic articles, please refer to Table 1 for detailed information. Our findings, presented through a forest plot in Fig 3, highlight the pooled sensitivity for mortality prediction from the prognostic studies as 0.78 [95%-CI: 0.71–0.85], accompanied by a pooled specificity of 0.78 [95%-CI: 0.73–0.84]. Furthermore, studies elucidating cfDNA’s diagnostic capability for sepsis reported a pooled sensitivity of 0.81 [95%-CI: 0.76–0.86] and specificity of 0.87 [95%-CI: 0.67–0.97], as shown in Fig 4. SROC curves for both diagnostic and prognostic studies were graphically depicted in S2 Fig in S1 File.

**Table 1:** Predictive analysis of sepsis-related mortality based on cfDNA cut-off concentration.

Study	Outcome	AUC (95%-CI)	Cutoff	Sensitivity	Specificity	LR+	LR−
Rannikko/2018/Finland [73]	7-day mortality	0.73 (0.69–0.85)	1690 ng/ml	70.4%	76.4%	2.99	0.39
Dwivedi/2012/Canada [25]	ICU mortality	0.97 (0.93–1.00)	2350 ng/ml	87.9%	93.5%	14	0.13
Rhodes/2006/UK [62]	ICU mortality^1^	0.84 (0.71–0.97)	127 ng/ml	92%	80%	4.5	0.1
Kung/2012/Taiwan [54]	Hospital mortality^2^	0.864 (0.773–0.984)	1012 ng/ml	82%	82%	4.58	0.22
Gau/2019/China [48]	28-day death	0.932 (0.787–1.00)	266.81 μg/L	90.9%	75.0%	3.64	0.12
Saukkonen/2008/Finland [88]	ICU mortality^3^	0.71 (0.62–0.80)	12000 GE/mL	67%	67%	2.03	0.49
Moreira/2010/Spain [58]	mortality	0.77	36000 GE/mL	75%	71%	2.6	0.4
Huang/2018/China [52]	Mortality	0.961 (0.93–0.992)	-	100%	81.43%	5.39	0
Duplessis/2018/USA [71]	28-day mortality	0.61 (0.46–0.75)	-	-	-	-	-
Avriel/2014/Israel [70]	28-day mortality	0.79 (0.68–0.90)	-	-	-	-	-
**Mortality in bacteremia patients**							
Huttunen/2011/Finland [42]	30-day mortality	0.81 (0.69–0.94)	1520 ng/ml	83%	79%	3.95	0.22
Forsblom/2014/Finland [41]	90-day mortality	0.71 (0.57–0.84)	1.99 μg/ml	67%	77%	2.91	0.43

1The median length of ICU stay was 5 (2–12) days

2 The median length of hospital stay was 14 days (range, 9–30 days)

3 The median length of ICU stay was 6.1 days

All the details about the extracted raw data from the studies are presented in S5-S7 Tables in S1 File.

### Quality assessment

Of the 28 diagnostic studies assessed using the QUADAS-2 tool (S3 Fig in S1 File), 21 exhibited a low risk of bias in participant selection.

Four studies were identified that focused primarily on the prognostic aspects of cfDNA in sepsis. These studies were assessed for risk of bias using the QUAPAS tool. Details of the quality assessment are presented in S8 and S9 Tables in S1 File.

### Publication bias assessment

S4 Fig in S1 File displays a funnel plot depicting the relationship between standard error and point estimate for the included articles. Egger’s test indicated the presence of publication bias (p = 0.001). Subsequently, the trim-and-fill method was employed to identify unpublished studies.

## Discussion

Sepsis, a common and potentially fatal condition, involves an excessive immune response that, if untreated, can progress to septic shock and death [3]. Research has explored cfDNA as a promising diagnostic and prognostic tool. This relatively new approach, derived from bodily fluids through natural processes like necrosis, apoptosis, active DNA release, or NETosis (suggested mechanism among septic patients [74]) [75], as well as pathological mechanisms like cellular turnover [76, 77], offers alternative diagnostic insights [78].

This systematic review provides compelling evidence supporting the use of cfDNA as a diagnostic tool in clinical settings for individuals at risk of sepsis and as a prognostic indicator for the outcome of complications. The findings reveal a significant elevation in cfDNA levels among septic patients compared to those without sepsis, and notably higher levels in patients who succumbed to sepsis in contrast to survivors. Beyond the primary scope of this review, we included some articles that examined the role of cfDNA in bacteremia. This addition was warranted given the potential for bacteremia to escalate into sepsis and septic shock.

Moving to details, a consistent elevation in circulating cfDNA levels was observed in septic patients compared to various control groups, including healthy individuals [25, 27, 44, 46–48, 51–56, 58–62, 64, 66], non-septic patients residing in ICUs [27, 45, 48, 50, 57, 62, 65, 79], and patients who underwent surgery without exhibiting signs of sepsis [49, 63]. Notably, this elevation in cfDNA levels was not restricted to adults but was also evident in pediatric populations, with septic children demonstrating a tendency towards higher cfDNA levels compared to their control groups [67–69].

Similar results were observed in other conditions. For instance, Hampson et al. observed elevated plasma cfDNA levels in burn patients, especially during septic episodes, with significantly higher levels in septic burn-injured patients compared to non-septic patients. These results emphasize the potential of cfDNA as a promising diagnostic marker for sepsis in this specific population [80]. Similarly, Lögters et al. found elevated levels of cfDNA/NETs in synovial fluid samples of septic arthritis patients when compared to noninfectious joint inflammation or osteoarthritis [81]. Further, Lehmann-Werman et al. found that the levels of hepatocyte-derived cfDNA were significantly higher in sepsis patients compared to healthy controls, indicating that the hepatocyte-derived cfDNA has potential applications in monitoring liver damage [82].

Moreover, individuals experiencing surgical sepsis exhibit a marked increase in the levels of cfDNA, encompassing both nuclear DNA (nuDNA) and mitochondrial DNA (mtDNA). Hawkins et al. have elucidated a noteworthy association between elevated nuDNA copy numbers and the initiation of chronic critical illness, with consequential implications for short-term clinical outcomes [83]. However, no statistically significant difference was found in overall cfDNA levels among hematological patients with febrile neutropenia, regardless of whether they developed complications such as sepsis or septic shock.

Interestingly, it was found that the cfDNA/leukocyte ratio specifically in acute myeloblastic leukemia patients was associated with the development of sepsis or septic shock [84]. Correspondingly, Alkhamis et al. showed CRP’s superior predictive efficacy for sepsis in postoperative patients compared to cfDNA, which did not increase notably before sepsis onset [85]. Consistent findings were observed among neonates, where cfDNA failed to predict sepsis before its onset [86].

Moreover, several studies have explored the diagnostic potential of cfDNA in sepsis by evaluating its ability to distinguish sepsis from SIRS using model building and AUC estimation. These studies have shown that cfDNA frequently outperforms other established clinical and laboratory parameters, including the SOFA score [27, 57]. Comparisons of cfDNA to procalcitonin yielded mixed results, with some studies showing superior performance of cfDNA [51], others comparable performance [57], and a few lower diagnostic accuracy [72]

Furthermore, our pooled analysis revealed significantly elevated cfDNA levels in deceased patients compared to survivors, highlighting the potential of cfDNA as a prognostic marker, particularly for mortality prediction [25, 27, 45, 48, 51, 54–56, 62, 63, 70–73, 87]. Notably, two studies did not reach statistical significance [63, 71]. Intriguingly, our findings further extend the prognostic utility of cfDNA beyond mortality prediction, demonstrating its ability to predict the course of sepsis and evaluate its severity. Notably, as sepsis severity increased, there was a corresponding rise in the concentration of cfDNA [52, 58, 60, 71].

Consistent with the above findings, Saukkonen et al. reported elevated cfDNA levels in non-surviving sepsis patients in both hospital and ICU settings, corroborating the prognostic potential of cfDNA in sepsis [88]. The prognostic utility of cfDNA was investigated by Chornenki et al. among sepsis and trauma patients. They discovered a positive correlation between cfDNA levels and sepsis severity, but not with trauma indicating its potential as a reliable prognostic biomarker for sepsis compared to trauma [74].

Several studies have demonstrated that cfDNA exhibits superior predictive performance compared to the APACHE II score in forecasting mortality among patients with severe sepsis. Furthermore, the integration of cfDNA with APACHE II has been proposed to enhance the accuracy of mortality prediction [25, 52, 70]. An alternative predictive model by Rannikko et al. indicated a 20-fold increase in the risk of death when combining high cfDNA levels with qSOFA ≥ 2 [73]. However, although Duplessis et al. demonstrated modest prognostic utility of cfDNA in predicting sepsis patient survival, they also reported conflicting findings regarding its prognostic accuracy compared to APACHE II. [71].

Extending our discussion to bacteremia, Urosevic et al. found that individuals hospitalized with bacteremia exhibited substantially elevated plasma cfDNA levels compared to uninfected individuals [43]. This finding aligns with the prognostic potential of plasma cfDNA, as demonstrated by Forsblom et al., who found that cfDNA levels accurately predicted mortality among patients with Staphylococcus aureus bacteremia treated in the ICU [41]. Notably, Huttunen et al. observed a significant difference in cfDNA concentrations between survivors and non-survivors during the first four days after a positive blood culture, further supporting the prognostic utility of cfDNA in bacteremia [42].

Although no studies have directly compared bacteremia with septic patients, we performed an analysis using unpaired T-tests revealed significant differences in mean cfDNA levels between bacteremia survivors [42] and survivors of sepsis [56, 73], with p-values of 0.0001 and 0.0006, respectively. These results highlight the potential of cfDNA in distinguishing between typical infectious and septic courses, warranting further investigation. The studies were selected for comparison due to the similarity of their methodologies.

Further exploration of cfDNA-neutralizing agents as therapeutics for severe sepsis is warranted. Nanoparticles have demonstrated promise as scavengers of inflammatory circulating cfDNA, effectively reducing inflammation and offering a promising avenue for treating this severe condition [89].

Noteworthy, there is a suggestion that during severe systemic inflammation, cfDNA is released initially by hematopoietic cells and later by nonhematopoietic cells [90], we may assume that lower levels of hematopoietic cells might reduce cfDNA levels. However, in immunocompromised individuals (e.g., HIV/AIDS, cancer, or autoimmune diseases), cfDNA levels may vary compared to healthy individuals due to the complex nature of contributing factors including the type and stage of the underlying disease, as well as specific treatments received (e.g., chemotherapy, immunosuppressants) [76, 91]. As far as we know, no studies have directly compared cfDNA levels in septic patients with and without compromised immune systems. Therefore, targeted studies are necessary to understand the impact of immune status on cfDNA levels in sepsis.

Longitudinal studies suggest cfDNA could serve as an effective real-time biomarker for tracking sepsis, providing insights into disease progression and treatment outcomes [25]. Liu JP et al. found that while PCT and WBC levels decreased, giving a misleading impression of recovery, cfDNA levels stayed elevated, correlating more accurately with the patient’s actual worsening condition [92]. This demonstrates cfDNA’s potential for superior monitoring of septic shock and detection of disease progression, surpassing traditional inflammatory markers. Further kinetic research is warranted.

### Strengths and limitations

Our study builds upon and broadens the insights presented in a recently published systematic review that explored the role of cfDNA in sepsis [26]. While the previous review concentrated primarily on adult populations, our research extends the scope to encompass pediatric patients. We have also been able to include a more extensive array of studies, including non-English articles. Combined with the qualitative review of the cfDNA use in bacteremia, the generalizability of this systematic review is enhanced.

Still, it is crucial to acknowledge certain limitations. Firstly, due to the heterogeneity in cfDNA measuring units (ng/mL and genome equivalent GE/ml) and the lack of standardized cfDNA detection methods, our analysis was unable to establish an applicable cut-off value for both diagnostic and prognostic assessment of sepsis. This heterogeneity in measurement and detection techniques introduces inconsistencies in cfDNA quantification, hindering the establishment of a definitive cut-off value [93, 94]. Hence, there is a need for additional research to establish standardized measurement and detection protocols for cfDNA [95]. This effort aims to facilitate the creation of reliable and reproducible cut-off values or establish a normal range for cfDNA in the healthy population. Moreover, the definition of sepsis has evolved over the years with changes in the criteria of diagnosis. This might increase the risk of selection bias in this meta-analysis. Some articles only presented data graphically without numerical details, which may impact the analysis. It is advisable to publish the data publicly for better transparency.

Lastly, because the fragments of human cfDNA carry insights into cellular oxidative stress, the timing of cfDNA may severely affect the predictability of the severity of disease. An example of how this affects disease modeling is provided by Jing et al., who had nearly 100% predictability of sepsis but a mere 80% of correctly predicting the clinical outcomes [27].

## Conclusion

Early diagnosis and prognosis of bloodstream infections remain critical challenges in clinical practice. Our meta-analysis has demonstrated a significant elevation in cfDNA levels in sepsis patients compared to healthy individuals, with further differentiation between survivors and non-survivors. Notably, these differences persisted irrespective of the primary infection source. While some studies have suggested diagnostic and intervention thresholds for cfDNA levels, further research is warranted to establish cfDNA as a standardized diagnostic tool for sepsis. Furthermore, integrating new laboratory techniques in machine learning models can provide more relevance and better predictability of patient outcomes.

## Supporting information

S1 File Supplementary figures and tables accompanied by the completed PRISMA checklist and the details of the quality assessment. (DOCX)

## List of abbreviations

AUC: Area under the curve
cfDNA: Cell-free DNA
ICU: Intensive care unit
SROC: Summary receiver operating characteristics
