# Supplementary material for: Role of cell-free DNA levels in the diagnosis and prognosis of sepsis and bacteremia: A systematic review and meta-analysis
Source: PLoS One. 2024 Aug 29;19(8):e0305895. doi: 10.1371/journal.pone.0305895 (PMC11361684; doi:10.1371/journal.pone.0305895)
Supplement: S1 File — (DOCX) [file pone.0305895.s001.docx]

**Role of cell-free DNA levels in the diagnosis and prognosis of sepsis and bacteremia: A systematic review and meta-analysis**

Mohammad Najm Dadam, MBBCh, Le Thanh Hien, MD, Engy M Makram, Lam Vinh Sieu, MD, Ahmad Morad, MBBCh, Nada Khalil, MBBCh, Linh Tran, PhD, Abdelrahman M Makram, MBBCh, MPH, Nguyen Tien Huy, MD, PhD

**Table of contents**

**Supplemental Figure 1.**  Forest plots for comparison of standardized mean difference (SMD) of cfDNA between sepsis and non-sepsis groups.

**Supplemental Figure 2.** SROC plots of the diagnostic and prognostic utility of cfDNA in septic patients.

**Supplemental Figure 3.** QUADAS-2 diagram for assessing the diagnostic studies.

**Supplemental Figure 4.** Funnel plot for evaluation of publication bias in the 28 diagnostic included studies.

**Supplemental Table 1.** Preferred Reporting Items for Systematic Reviews and Meta-Analyses (PRISMA Checklist).

**Supplemental Table 2.** Search term details for each database.

**Supplemental Table 3.** Characteristics of studies included in the meta-analysis.

**Supplemental Table 4.** Characteristics of included articles in the qualitative review.

**Supplemental Table 5.** Comparison of diagnostic mean and standard deviation (SD) between Groups.

**Supplemental Table 6.** Comparison of prognostic mean and standard deviation (SD) between survivors and non-survivors.

**Supplemental Table 7.** Comparison of prognostic mean and standard deviation (SD) between septic patients and severe septic patients.

**Supplemental Table 8.** Quality assessment of the included studies using the QUADAS-2 tool.

**Supplemental Table 9.** Quality assessment of the included studies using the QUAPAS tool.

**Supplemental Figure 1.**  Forest plots for comparison of standardized mean difference (SMD) of cfDNA between sepsis and non-sepsis groups.


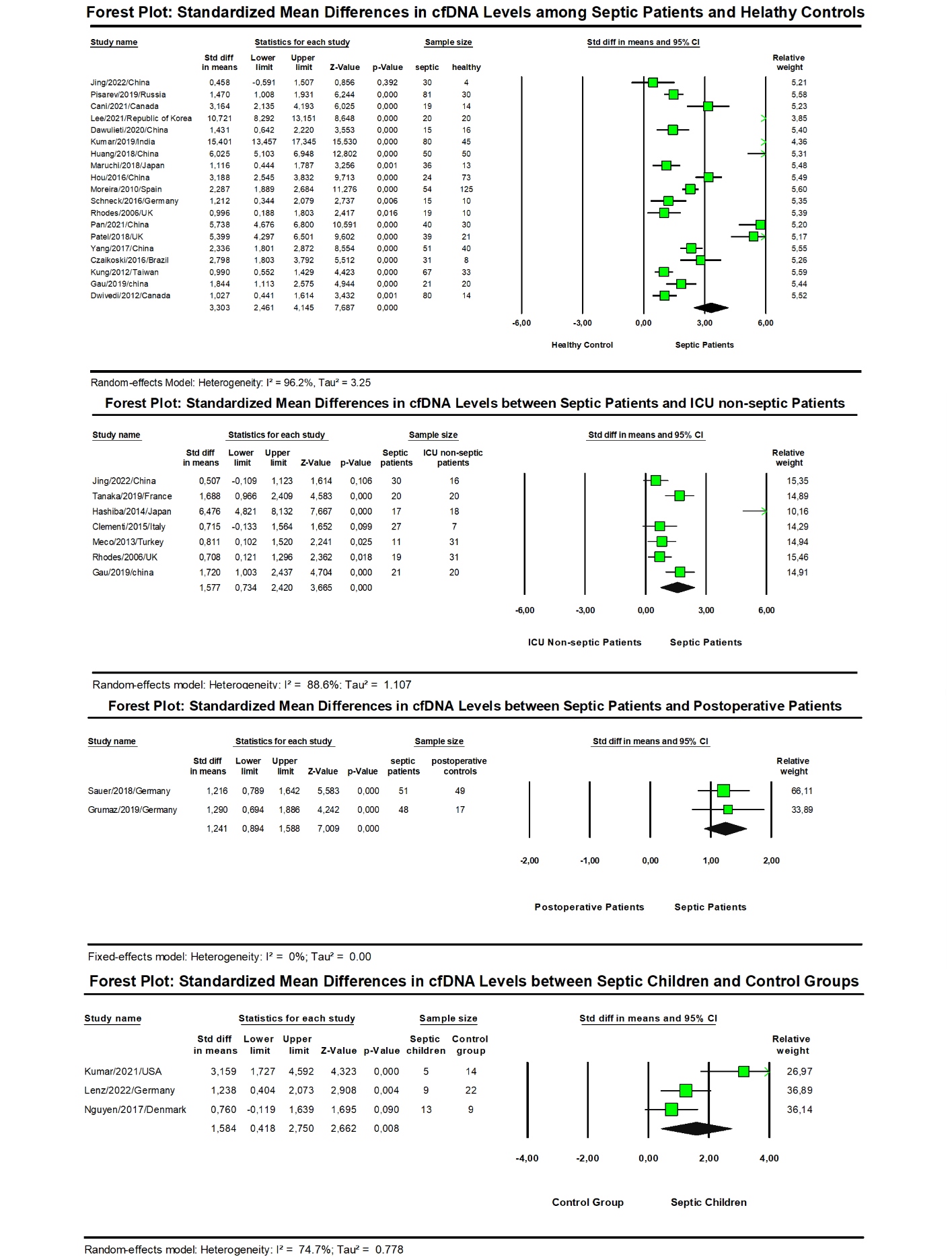


**Supplemental Figure 2.** SROC plots of the diagnostic and prognostic utility of cfDNA in septic patients.


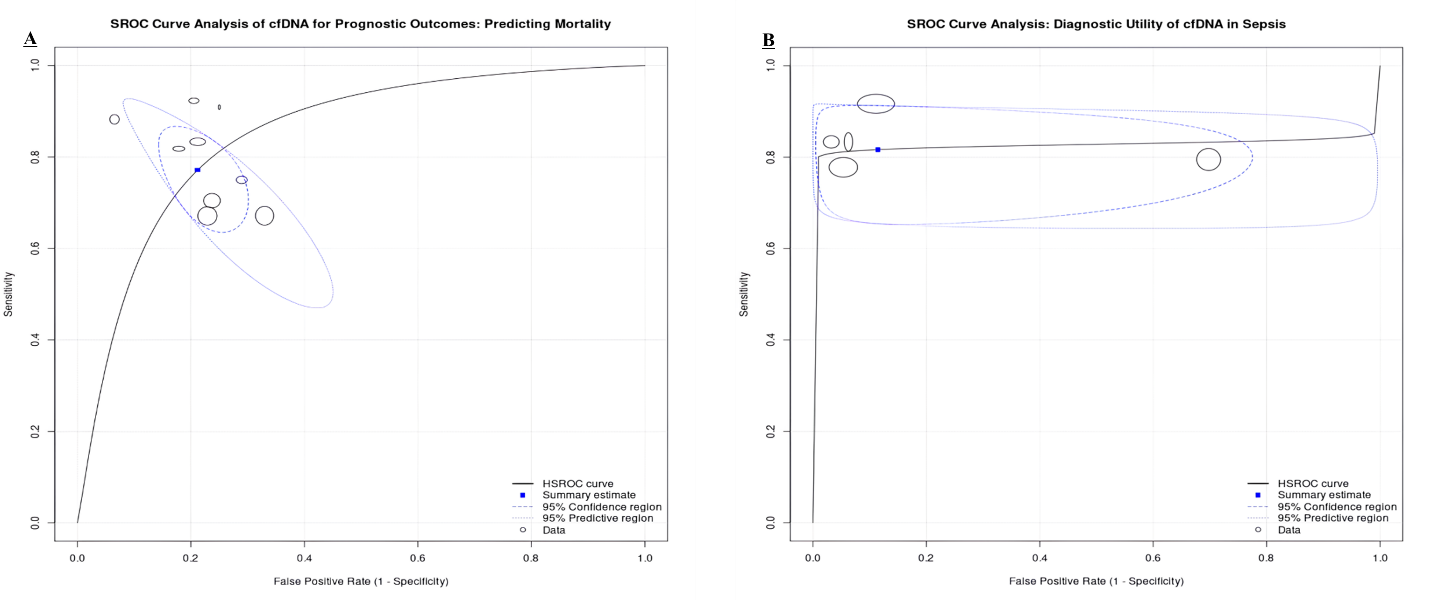


**Supplemental Figure 3.** QUADAS-2 diagram for assessing the diagnostic studies.


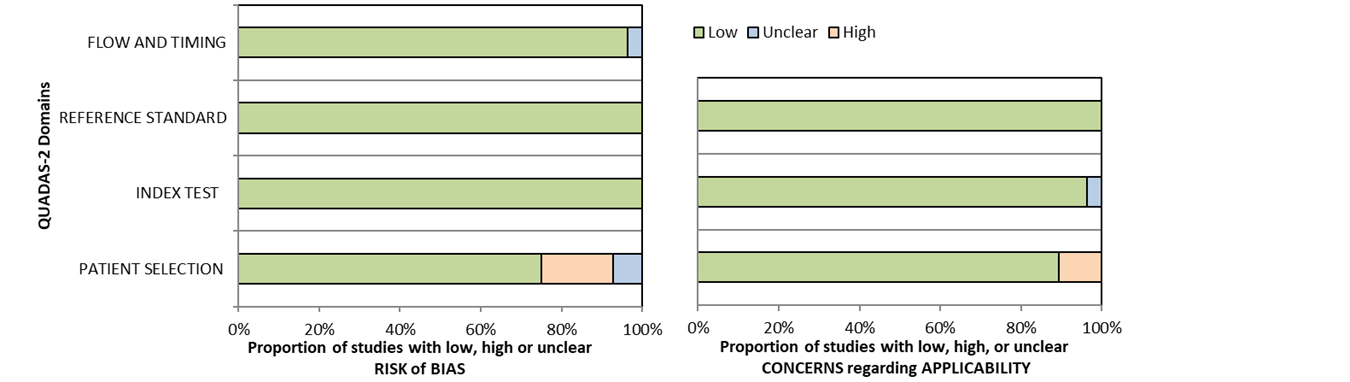


**Supplemental Figure 4.** Funnel plot for evaluation of publication bias in the 28 diagnostic included studies.


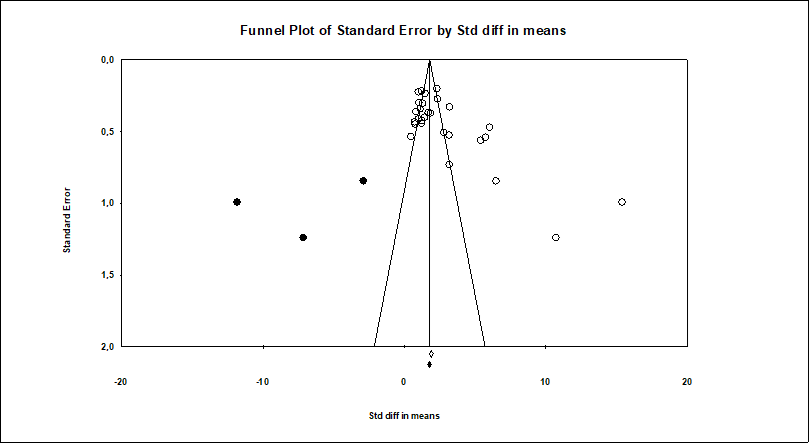


**Supplemental Table 1.** Preferred Reporting Items for Systematic Reviews and Meta-Analyses (PRISMA Checklist).

| **Section/topic** | **#** | **Checklist item** | **Reported on page #** |
| --- | --- | --- | --- |
| **TITLE** | | |  |
| Title | 1 | Identify the report as a systematic review, meta-analysis, or both. | 1 |
| **ABSTRACT** | | |  |
| Structured summary | 2 | Provide a structured summary including, as applicable: background; objectives; data sources; study eligibility criteria, participants, and interventions; study appraisal and synthesis methods; results; limitations; conclusions and implications of key findings; systematic review registration number. | 2 |
| **INTRODUCTION** | | |  |
| Rationale | 3 | Describe the rationale for the review in the context of what is already known. | 3 |
| Objectives | 4 | Provide an explicit statement of questions being addressed with reference to participants, exposures, comparisons, outcomes, and study design (PICOS). | 4 |
| **METHODS** | | |  |
| Protocol and registration | 5 | Indicate if a review protocol exists, if and where it can be accessed (e.g., Web address), and, if available, provide registration information including registration number. | 4 |
| Eligibility criteria | 6 | Specify study characteristics (e.g., PICOS, length of follow-up) and report characteristics (e.g., years considered, language, publication status) used as criteria for eligibility, giving rationale. | 4 |
| Information sources | 7 | Describe all information sources (e.g., databases with dates of coverage, contact with study authors to identify additional studies) in the search and date last searched. | 4 |
| Search | 8 | Present full electronic search strategy for at least one database, including any limits used, such that it could be repeated. | 4 |
| Study selection | 9 | State the process for selecting studies (i.e., screening, eligibility, included in systematic review, and, if applicable, included in the meta-analysis). | 5 |
| Data collection process | 10 | Describe method of data extraction from reports (e.g., piloted forms, independently, in duplicate) and any processes for obtaining and confirming data from investigators. | 5 |
| Data items | 11 | List and define all variables for which data were sought (e.g., PICOS, funding sources) and any assumptions and simplifications made. | NA |
| Risk of bias in individual studies | 12 | Describe methods used for assessing risk of bias of individual studies (including specification of whether this was done at the study or outcome level), and how this information is to be used in any data synthesis. | 6 |
| Summary measures | 13 | State the principal summary measures (e.g., risk ratio, difference in means). | 5 |
| Synthesis of results | 14 | Describe the methods of handling data and combining results of studies, if done, including measures of consistency (e.g., I^2^) for each meta-analysis. | 5 |
| Risk of bias across studies | 15 | Specify any assessment of risk of bias that may affect the cumulative evidence (e.g., publication bias, selective reporting within studies). | 5 |
| Additional analyses | 16 | Describe methods of additional analyses (e.g., sensitivity or subgroup analyses, meta-regression), if done, indicating which were pre-specified. | 5 |
| **RESULTS** | | |  |
| Study selection | 17 | Give numbers of studies screened, assessed for eligibility, and included in the review, with reasons for exclusions at each stage, ideally with a flow diagram. | 25 |
| Study characteristics | 18 | For each study, present characteristics for which data were extracted (e.g., study size, PICOS, follow-up period) and provide the citations. | 22-25 |
| Risk of bias within studies | 19 | Present data on risk of bias of each study and, if available, any outcome level assessment (see item 12). | 8 |
| Results of individual studies | 20 | For all outcomes considered (benefits or harms), present, for each study: (a) simple summary data for each intervention group (b) effect estimates and confidence intervals, ideally with a forest plot. | 26-27 |
| Synthesis of results | 21 | Present results of each meta-analysis done, including confidence intervals and measures of consistency. | 7 |
| Risk of bias across studies | 22 | Present results of any assessment of risk of bias across studies (see Item 15). | 8 |
| Additional analysis | 23 | Give results of additional analyses, if done (e.g., sensitivity or subgroup analyses, meta-regression [see Item 16]). | 7 |
| **DISCUSSION** | | |  |
| Summary of evidence | 24 | Summarize the main findings including the strength of evidence for each main outcome; consider their relevance to key groups (e.g., healthcare providers, users, and policy makers). | 9-11 |
| Limitations | 25 | Discuss limitations at study and outcome level (e.g., risk of bias), and at review-level (e.g., incomplete retrieval of identified research, reporting bias). | 11 |
| Conclusions | 26 | Provide a general interpretation of the results in the context of other evidence, and implications for future research. | 12 |
| **FUNDING** | | |  |
| Funding | 27 | Describe sources of funding for the systematic review and other support (e.g., supply of data); role of funders for the systematic review. | NA |

**Supplemental Table 2.** Search term details for each database.

| **Databases** | **Search terms** | **Initial search** | **Updated search** |
| --- | --- | --- | --- |
| **PubMed** | (sepsis OR septic OR septicemia OR septicemias) AND DNA AND (free OR circulating OR circulation) AND (prediction OR predictions OR predict OR predictive OR predicted OR prognosis OR prognostic OR forecast OR diagnostic OR diagnosis) | 457 | 118 |
| **Scopus** | (sepsis OR septic OR septicemia OR septicemias) AND DNA AND (free OR circulating OR circulation) AND (prediction OR predictions OR predict OR predictive OR predicted OR prognosis OR prognostic OR forecast OR diagnostic OR diagnosis) | 238 | 191 |
| **Science direct** | (sepsis OR septic OR septicemia OR septicemias) AND (DNA) AND (Free OR plasma OR circulation) | 100 | 68 |
| **Google scholar** | a- with all of the words: DNA Free  with at least one of the words: sepsis septic septicemia septicemias  limit: in the title of the article - 43  b-with all of the words: DNA circulating  with at least one of the words: sepsis septic septicemia septicemias  limit: in the title of the article - 21  c- with all of the words: DNA plasma  with at least one of the words: sepsis septic septicemia septicemias  limit: in the title of the article - 38  d- with all of the words: DNA circulation  with at least one of the words: sepsis septic septicemia septicemias  limit: in the title of the article - 0 | 102 | 29 |
| **Cochrane** | (sepsis OR septic OR septicemia OR septicemias) AND DNA AND (free OR circulating OR circulation) | 43 | 43 |
| **Clinicaltrials.gov** | Disease: sepsis OR septic OR septicemia OR septicemias  Other term: (DNA) AND (free OR circulating OR plasma OR circulation) | 45 |  |
| **Web of science** | (sepsis OR septic OR septicemia OR septicemias) AND DNA AND (free OR circulating OR circulation) AND (prediction OR predictions OR predict OR predictive OR predicted OR prognosis OR prognostic OR forecast OR diagnostic OR diagnosis) | 178 | 105 |
| **VHL** | (sepsis OR septic OR septicemia OR septicemias) AND DNA AND (free OR circulating OR circulation) AND (prediction OR predictions OR predict OR predictive OR predicted OR prognosis OR prognostic OR forecast OR diagnostic OR diagnosis) | 217 | 6 |
| **WHO ICTRP** | In the title: (sepsis OR septic OR septicemia OR septicemias) AND DNA | 5 | 0 |
| **mRCT** | (sepsis OR septic OR septicemia OR septicemias) AND DNA | 12 | NA |
| **Embase** | (sepsis OR septic OR septicemia OR septicemias) AND DNA | 842 | 875 |
| **SIGLE** | (sepsis OR septic OR septicemia OR septicemias) AND DNA | 6 | 0 |
| **WHO GHL** | (sepsis OR septic OR septicemia OR septicemias) AND DNA AND (free OR circulating OR circulation) | 15 | 6 |

NA: Not applicable as the database was discontinued.

**Supplemental Table 3.** Characteristics of studies included in the meta-analysis.

| Studies included in the quantitative analysis | | | | | | | | | | |
| --- | --- | --- | --- | --- | --- | --- | --- | --- | --- | --- |
| **ID Reference** | **Sample size** | **Age (Years)**  **Mean (SD)** | **Gender M/F** | **Follow-up duration** | **Survival/Deaths** | **Sepsis definition** | **Sepsis score** | **Patients setting** | **Days of collection** | **Method of detection** |
| Dwivedi/2012/Canada (1) | Sepsis 80 | Survivors 60 (14.9) | 55/25 | 28 days | 46/34 | ACCP/SCCM Consensus | APACHE II & MODS | ICU admitted | 1-7,14,21,28 | Spectrophotometer |
|  |  | Deceased 68(13.4) |  |  |  |  |  |  |  |  |
| Rannikko/2018/Finland (2) | Sepsis 481 | 61.75 (22.8) | 253/228 | 28 days | 384/97 | Sepsis-2 | qSOFA | Emergency department admitted | 0,1,2,3,4 | QUBIT® 2.0 Fluorometer with  Quant-iT PicoGreen dsDNA |
| Maruchi/2018/Japan (3) | Sepsis 55 | 67 (18) | 39/16 | 28 days | 33/18 | ACCP/SCCM Consensus | APACHE II & SOFA | ICU admitted | 1, 3, 7 | QUBIT® 2.0 Fluorometer with  Quant-iT PicoGreen dsDNA |
|  | Healthy 13 | 60 (18.2) | 8/5 |  |  |  |  |  |  |  |
| Hashiba/2014/Japan(4) | Sepsis 17 | 57 (42.8) | 10/7 | NR | NR | ACCP/SCCM Consensus | APACHE II & SOFA | ICU admitted. | 24 h after diagnosis of sepsis. | QUBIT® 2.0 Fluorometer with  Quant-iT PicoGreen dsDNA |
|  | ICU non-sepsis 18 | 52 (48.2) | 8/10 |  |  |  |  |  |  |  |
| Montero/2014/Spain (5) | Sepsis 117 | 62 (15.7) | 65/52 | NR | 76/41 | ACCP/SCCM Consensus | APACHE II & SOFA | ICU admitted. | At admission and after 24 hours | qPCR assay for the β-globin gene |
|  | SIRS 43 | 62 (20.7) | 14/29 |  |  |  |  |  |  |  |
| MEÇO/2013/Turkey (6) | Sepsis 11 | 51.1 (18.7) | 22/20 | 28-day | 31/11 | ACCP/SCCM Consensus | APACHE II, SOFA, SAPS | Ventilated ICU patients | At admission | qPCR assay for the β-globin gene |
|  | Non-septic 31 |  |  |  |  |  |  |  |  |  |
| Rhodes/2006/UK (7) | Sepsis 52  Controls 10 | Survivors 60.25 (16)  Deceased 66.25 (13) | 32/20 | 3 months | 39/13 | ACCP/SCCM Consensus | SOFA | ICU admitted | At admission | qPCR assay for the β-globin gene |
| Huang/2018/China (8) | Sepsis 50  Severe sepsis 55 | 57.6 (14.8) | 54/51 | Median ICU stay 6 days | NR | Chinese guidelines for the management of severe sepsis and septic shock 2014 | APACHE II  SOFA | Emergency center admitted | At ICU admission | Magnetic bead method |
|  | Healthy 50 | 56.0 (15.3) | 25/25 |  |  |  |  |  |  |  |
| Moreira/2010/Spain (9) | Sepsis 54^[[1]](#footnote-1)^  Healthy 125 | 44 (20) | whole sample^[[2]](#footnote-2)^ 66/44  Septic NR | NR | From septic patients 38/16 | ACCP/SCCM Consensus | SOFA | Emergency department, ICU, or ward admitted | At diagnosis time | qPCR of the β-globin gene |
| Schneck/2016/Germany (10) | Sepsis 15 | 61 (13.26) | 8/7 | 28 days | 9/6 | Surviving Sepsis Campaign | APACHE II, SOFA, SAPS II | Surgical ICU admitted | 0, 1, 3, 7 | qPCR assay for the β-globin gene |
|  | Healthy 10 | 58.2 (14.5) |  |  |  |  |  |  |  |  |
| Avriel/2014/Israel (11) | 108 Septic | 55.88 (17.2) | 71/37 | 28 days | 73/35 | ACCP/SCCM Consensus | APACHE II | ICU admitted | Within 12 hours of admission | Fluorometer with SYBR® Stain |
| Duplessis/2018/USA (12) | 203 including 24 SIRS patients | Survival 53.6 (20.1)  Deceased 64.3 (19.1) | 115/88 | 28 days | 190/13  All mortality cases were septic. | ACCP/SCCM Consensus | APACHE II | Emergency department | At admission and 24 hours later | Fluorometer with SYBR® Stain |
| Clementi/2015/Italy (13) | Sepsis 27  ICU non-septic 7 | 62 (18) | 19/8 | 8 days^[[3]](#footnote-3)^ | 16/11 | Surviving Sepsis Campaign | SOFA | ICU admitted | At ICU admission | Real-time qPCR  for the β-actin gene |
| Hou/2016/China (14) | Sepsis 24 | 58.3 (12.7) | 15/9 | NR | 15/9 | SIRS with positive blood culture. | APACHE II | ICU admitted | In 6 hours after admission | Multilabel plate reader (VECTOR 3) |
|  | Healthy 73 | 52.6 (12.9) | 45/28 |  |  |  |  |  |  |  |
| Jing/2022/China (15) | Sepsis 30 | 64.9 (9.9) | 28/22 | 28 days | 20/10 | Sepsis-3 | SOFA, APACHI II | ICU admitted | 0, 1, 2, 3, and 4-7 | Qubit 3.0 fluorometer |
|  | ICU non-septic 16 | 55.6 (10.7) |  |  |  |  |  |  |  |  |
|  | Healthy 4 | 37.75 (2.27) |  |  |  |  |  |  |  |  |
| Pisarev/2019/Russia (16) | Sepsis 81 | 56 (14.3) | 52/29 | 30 days | NR | Sepsis-3 | SOFA | ICU admitted | NR | spectrofluorometer with PicoGreen |
|  | Healthy 30 |  |  |  |  |  |  |  |  |  |
| Tanaka/2019/France (17) | Sepsis 20 | 68 (13.9) | 17/3 | 28 days | 15/5 | Surviving Sepsis Campaign | SOFA, SAPS II | ICU admitted | on days 1, 2, and 7 | Quantit™ Picogreen dsDNA Reagent |
|  | ICU non-sepsis 20 | 66.7 (17) | 13/7 |  | 18/2 |  |  |  |  |  |
| Dawulieti/2020/China (18) | Sepsis 15 | NR | NR | NR | NR | Sepsis-3 | NR | Hospital admitted | NR | Quant-iT PicoGreen double-stranded DNA Assay Kit |
|  | Healthy 16 |  |  |  |  |  |  |  |  |  |
| Grumaz/2019/Germany (19) | Sepsis 48 | 66.8 (10.3) | 37/11 | 47 days | 39/9 | Surviving Sepsis Campaign | SOFA, APACHE II SAPS II | ICU admitted | 0, 1, 2, 7, 14, 21, 28 | NGS |
|  | Control 17 | 65 (8.9) | 10/7 |  |  |  |  |  |  |  |
| Cani/2021/Canada (20) | Sepsis 19 | 62.3 (19.2) | 10/9 | 14 days | 13/6 | Sepsis-3 | SOFA | ICU admitted with pneumonia | Within 24 hours of meeting sepsis criteria | Quant-iT PicoGreen double-stranded DNA Assay Kit. |
|  | healthy 14 | 58.3 (7.4) | 6/8 |  |  |  |  |  |  |  |
| Lee/2021/Republic of Korea (21) | Covid-19 sepsis 20 | NR | NR | NR | NR | Sepsis-3 | - | Hospital admitted | NR | NanoDrop spectrophotometry |
|  | Healthy 20 |  |  |  |  |  |  |  |  |  |
| Kumar/2019/India (22) | Sepsis 80 | 49 (17) | 58/22 | NR | NR | Surviving Sepsis Campaign | NR | SIRS |  | Fluorometer with SytoxGreen dye |
|  | Healthy 45 | 40 (11) | 34/11 |  |  |  |  |  |  |  |
| Pan/2021/China (23) | Sepsis 40 | 47.3(12.37) | 32/8 | 28 days^[[4]](#footnote-4)^ | Septic 33/7 | Sepsis-3 | SOFA | Acute pancreatitis | At diagnosis | PicoGreen assay kit |
|  | Non septic 30 | 55.8(10.76) | 22/8 |  |  |  |  |  |  |  |
|  | Healthy 30 | 50.9 | 21/9 |  |  |  |  |  |  |  |
| Patel/2018/UK (24) | Sepsis 39 | 75 (15.3) | 20/19 | 30-day^[[5]](#footnote-5)^ | 35/4 | Surviving Sepsis Campaign Guidelines | SOFA, APACHE II | Hospital admitted | At admission, 4, 7 | Fluorometer with SytoxGreen stain |
|  | Severe sepsis 60 | 72.3 (18.2) | 41/19 |  | 48/12 |  |  |  |  |  |
|  | Sepsis shock 13 | 71(8.7) | 10/3 |  | 6/7 |  |  |  |  |  |
|  | Healthy 21 | 64(21.5) | 13/8 |  | 21/0 |  |  |  |  |  |
| Yang/2017/China (25) | Sepsis 52 | 53.3 (14.5) | 32/20 | NR | NR | Surviving Sepsis Campaign  Guidelines | APACHE II | ICU admitted | At ICU admission, after 36 h | Fluorometer with SytoxGreen stain |
|  | Healthy 40 | 51.8 (13.2) | 24/16 |  |  |  |  |  |  |  |
| Sauer/2018/Germany (26) | Sepsis 51 | 63 (15) | 39/12 | 7 days | NR | ACCP/SCCM Consensus | SOFA, APACHE II | ICU admitted | 0, 3, 7 days | fluorescent assay |
|  | control 49 | 66.3 (5.3) | 34/15 |  |  |  |  |  |  |  |
| Czaikoski/2016/Brazil (27) | Sepsis 31 | 58.54 | 17/14 | NR | 12/19 | ACCP/SCCM Consensus | SOFA, APACHE II | Emergency Department | NR | Fluorometer with  Quant-iT PicoGreen ® kit |
|  | Healthy 8 | - |  |  |  |  |  |  |  |  |
| Kung/2012/Taiwan (28) | Sepsis 67 | 64.8 (13.8) | 44/23 | 14^[[6]](#footnote-6)^ | 56/11 | ACCP/SCCM Consensus | SOFA  APACHE II | Emergency Department and ICU | At admission, 4 and 7 | real-time qPCR assay for β-globin and MT-ND2 genes |
|  | Healthy 33 | 66.3 (8) | 22/11 |  |  |  |  |  |  |  |
| Gau/2019/china (29) | Sepsis 36^[[7]](#footnote-7)^ | 57.8 (13) | 22/14 | 28 days | 4/11^[[8]](#footnote-8)^ | Sepsis-3 | SOFA  APACHE II | ICU admitted | At admission | Fluorometer with  PicoGreen kit |
|  | ICU non-sepsis 20 | 55.7 (17.1) | 14/6 |  |  |  |  |  |  |  |
|  | Healthy 20 | 49.4 (13.2) | 13/7 |  |  |  |  |  |  |  |
| **Pediatric population** | | | | | | | | | | |
| Lenz/2022/Germany | EONS^[[9]](#footnote-9)^ 3 | 2 days (1) | 2/1 | NR | 1/2 | European Medical Agency (EMA) sepsis scoring | NR | neonatal intensive care unit | At admission before antibiotic treatment | Fluorometer with SytoxGreen stain |
|  | Controls 10 | 1.5 days (0.71) | 7/3 |  | 10/0 |  |  |  |  |  |
|  | LONS^[[10]](#footnote-10)^ 6 | 44.01 days (35.63) | 4/2 |  | 5/1 |  |  |  |  |  |
|  | Controls 12 | 25.58 days (22.72) | 7/5 |  | 12/0 |  |  |  |  |  |
| Nguyen/2017/Denmark | LOS 13 | 20.3 days (15.5) | NR | NR | NR | NR | SOFA | Neonatal ICU admitted | 1, 2, 3, 4, 5, 6 | Fluorometer with  Quant-iT PicoGreen ® kit |
|  | healthy 27 |  |  |  |  |  |  |  |  |  |
| Kumar/2021/USA | Sepsis 5 | NR | NR | NR | NR | NR | NR | inpatient units of the hospital | Once at admission | Qubit Fluorometer using dsDNA HS Assay |
|  | Healthy 14 | 6.8 (4.6) | 6/8 |  |  |  |  |  |  |  |

**Supplemental Table 4.** Characteristics of included articles in the qualitative review.

| **Studies Included in the Qualitative Review** | | | | | | | | | | |
| --- | --- | --- | --- | --- | --- | --- | --- | --- | --- | --- |
| **Articles focusing on bacteremia patients** | | | | | | | | | | |
| **ID Reference** | **Sample size** | **Age (Years)**  **Mean (SD)** | **Gender M/F** | **Follow-up duration** | **Survival/Deaths** | **Diagnosis** | **Scores** | **Patients setting** | **Days of collection** | **Method of detection** |
| Forsblom/2014/Finland (30) | 418 | 50% > 60 | 262/156 | 90 days | 345/73 | Positive blood culture | Pitt bacteremia score | From 12 hospitals | 3, 5 | Quant-iT™ high-sensitivity DNA assay kit and QubitH fluorometer |
| Huttunen/2011/Finland (31) | 132 | 58.2 (22.2) | 70/62 | 30 days | 118/18 | Positive blood culture | SOFA | Hospital admitted | 1–4 days after positive blood culture  on day 5–17  on recovery | QUBIT® Fluorometer with  Quant-iT PicoGreen dsDNA |
| Urosevic/2022/Australia (32) | Bacteremia 49 | 64.06 | 26/23 | NR | NR | Positive blood culture | qSOFA score | Hospital admitted | On admission | Qubit dsDNA HS Assay and a Qubit 2.0 Fluorometer |
|  | No paired BC 14 | 63.07 | 9/5 |  |  |  |  |  |  |  |
|  | Healthy 9 | 45 | 6/3 |  |  |  |  |  |  |  |
| **Articles focusing on septic patients** | | | | | | | | | | |
| **ID Reference** | **Sample size** | **Age (Years)**  **Mean (SD)** | **Gender M/F** | **Follow-up duration** | **Survival/Deaths** | **Sepsis definition** | **Sepsis score** | **Patients setting** | **Days of collection** | **Method of detection** |
| Alkhamis/2014/Croatia (33) | 115 | 63.04 (5.3) | 58/57 | 5 days | 111/4 | ACCP/SCCM consensus | APACHE III | Elective surgery patients | Day before surgery and day 1 | Real-time PCR |
| Chornenki/2019/Canada (34) | Trauma 77  Septic 49  Healthy 26 | 53.6 (24.2) | 56/21 | 28 days | 64/13 | ACCP/SCCM consensus | MODS | ICU admitted | NR | Spectrophotometer and qPCR |
| Lehmann-Werman/2018/USA (35) | Septic 56  Healthy 12 | NR | NR | NR | NR | NR | NR | NR | Before surgery and 12, 30, 95 after it | - Qbit double-strand molecular probes - digital droplet PCR assay for measurement of hepatocyte cfDNA |
| Abdelaal/2018/Egypt (36) | Septic 30 | 26 days (32.7)* | 15/15 | 5 days | 21/9 | NR | PELOD score^[[11]](#footnote-11)^ | ICU admitted | On admission, day 5 | qPCR assay for the β-globin gene |
| Purhonen/2015/Finland (37) | Septic AML 21^[[12]](#footnote-12)^ | 49.5 (14.995) | 61/39 | 3 days | 18/3^[[13]](#footnote-13)^ | ACCP/SCCM consensus | NR | adult hematology  ward | 0, 1, 3 | qPCR assay for the β-globin gene |
| Saukkonen/2008/Finland (38) | 255 | 61.9 (6.2) | 176/79 | NR | 188/67 | ACCP/SCCM consensus | APACHE II, SOFA & SAPS II | ICU admitted | 0, 3 | qPCR assay for the β-globin gene |
| Lögters/2009/Germany (39) | Septic Arthritis 9  Noninfectious arthritis 16 | 45.8 (2.6) | 3/6 | NR | NR | positive arthrocentesis culture / intraoperative findings  consistent with septic arthritis (e.g., frank pus) | NR | NR | Synovial fluid was obtained promptly after admission | qPCR assay for the β-globin gene, quantified by densitometry |
| Hampson/2017/UK (40) | Septic 35 | 42 (15,4) | 34/20 | 12 months | 14 out of the 57 remaining patients died. | Sepsis criteria according to American Burn Association (ABA), and  positive bacterial culture or clinical response to antibiotics | APACHE II, SOFA & MOF | Burns Centre admitted | 1, 3, 7, 14, 21, 28 days.  2,3, 6, 12 months. | qPCR assay for the cytochrome b and β-globin gene |
|  | Non-septic 22 | 34 (16,6) | 15/7 |  |  |  |  |  |  |  |
|  | Healthy 19 | 48 (31,2) | 8/11 |  |  |  |  |  |  |  |
| Martins/2000/Brazil (41) | Septic 11 | 51.3 (18.9) | 6/5 | NR | 6/5 | ACCP/SCCM Consensus | APACHE | ICU admitted | NR | Ethidium Bromide Fluorescence |

**Supplemental Table 5.** Comparison of diagnostic mean and standard deviation (SD) between Groups.

| **Study** | **Unit** | **Sepsis Mean** | **Sepsis SD** | **Sepsis sample size** | **Control** | **Control Mean** | **Control SD** | **Control sample size** |
| --- | --- | --- | --- | --- | --- | --- | --- | --- |
| Jing/2022/China (15) | ng/ml | 476,32 | 1086,45 | 30 | Healthy | 2,65 | 2,4 | 4 |
| Pisarev/2019/Russia (16) | ng/ml | 997,3 | 546,3 | 81 | Healthy | 277,2 | 280,8 | 30 |
| Cani/2021/Canada (20) | μg/ml | 5,3 | 1,4 | 19 | Healthy | 1,9 | 0,2 | 14 |
| Lee/2021/Republic of Korea (21) | μg/ml | 2,8 | 0,3 | 20 | Healthy | 0,4175 | 0,0936 | 20 |
| Dawulieti/2020/China (18) | ng/ml | 641,45 | 558,3 | 15 | Healthy | 84,22 | 47,45 | 16 |
| Kumar/2019/India (22) | ng/μl | 22,08 | 1,16 | 80 | Healthy | 7,04 | 0,5 | 45 |
| Huang/2018/China (8) | ng/ml | 1389,3 | 322,1 | 50 | Healthy | 16,8 | 5,72 | 50 |
| Maruchi/2018/Japan (3) | ng/ml | 2547,39 | 1475,64 | 36 | Healthy | 1080,569 | 648,5932 | 13 |
| Hou/2016/China (14) | ng/ml | 1426,54 | 863,79 | 24 | Healthy | 69,66 | 24,66 | 73 |
| Moreira/2010/Spain (9) | GE/ml | 40150 | 31464,5 | 54 | Healthy | 767,5 | 482,2 | 125 |
| Schneck/2016/Germany (10) | GE/ml | 301,45 | 315,3 | 15 | Healthy | 3,4 | 1,2 | 10 |
| Rhodes/2006/UK (7) | ng/ml | 206 | 237 | 19 | Healthy | 13,3 | 4,3 | 10 |
| Pan/2021/China (23) | ng/ml | 929,5 | 193 | 40 | Healthy | 84,6 | 27,2 | 30 |
| Patel/2018/UK (24) | ng/ml | 1396,6 | 300 | 39 | Healthy | 74 | 55 | 21 |
| Yang/2017/China (25) | ng/ml | 6386 | 3126 | 51 | Healthy | 893 | 292 | 40 |
| Czaikoski/2016/Brazil (27) | ng/ml | 1640 | 334,5 | 31 | Healthy | 782,1 | 132,3 | 8 |
| Kung/2012/Taiwan (28) | ng/ml | 597,3 | 700 | 67 | Healthy | 28,3 | 17 | 33 |
| Gao/2019/China (29) | ng/ml | 169,76 | 57,05 | 21 | Healthy | 92,23 | 14,26 | 20 |
| Dwivedi/2012/Canada (1) | ng/ml | 2640 | 1770 | 80 | Healthy | 930 | 760 | 14 |
| Jing/2022/China^[[14]](#footnote-14)^ (15) | ng/ml | 476,32 | 1086,45 | 30 | ICU non-septic | 28,97 | 40,6 | 16 |
| Rhodes/2006/UKa (7) | ng/ml | 206 | 237 | 19 | ICU non-septic | 92 | 88 | 31 |
| Gao/2019/Chinaa (29) | ng/ml | 169,76 | 57,05 | 21 | ICU non-septic | 97,66 | 13,45 | 20 |
| Tanaka/2019/France (17) | ng/ml | 1058,76 | 359,5 | 20 | ICU non-septic | 601,9 | 131,6446 | 20 |
| Hashiba/2014/Japan (4) | ng/ml | 966 | 198 | 17 | ICU non-septic | 65 | 26 | 18 |
| Clementi/2015/Italy (13) | GE/ml | 7731 | 11240 | 27 | ICU non-septic | 482 | 728 | 7 |
| Meco/2013/Turkey (6) | GE/ml | 64881,33 | 152906,3 | 11 | ICU non-septic | 2740 | 5964 | 31 |
| Sauer/2018/Germany (26) | ng/ml | 453,5 | 274,7 | 51 | Post-OP | 206,3 | 76,4 | 49 |
| Grumaz/2019/Germany (19) | ng/ml | 443 | 358 | 48 | Post-OP | 43,3 | 41,5 | 17 |
| Kumar/2021/USA (42) | ng/μl | 16,35 | 1,47 | 5 | healthy | 11,29 | 1,64 | 14 |
| Lenz/2022/Germany (43) | ng/ml | 6451,68 | 3975,28 | 9 | EMA negative | 3016,6 | 2146,4 | 22 |
| Nguyen/2017/Denmark (44) | ng/ml | 1500 | 769,6 | 13 | healthy | 1034,6 | 219,3 | 9 |

**Supplemental Table 6.** Comparison of prognostic mean and standard deviation (SD) between survivors and non-survivors.

| **Study** | **Unit** | **Survivors Mean** | **Survivors SD** | **Survivors sample size** | **Non-survivors Mean** | **Non-survivors SD** | **Non-survivors sample size** |
| --- | --- | --- | --- | --- | --- | --- | --- |
| Maruchi/2018/Japan (3) | ng/ml | 2582,816 | 1615,078 | 23 | 3053,83 | 2361,29 | 13 |
| Clementi/2015/Italy (13) | GE/ml | 3235,67 | 3919,4 | 16 | 8962 | 3599 | 11 |
| Hou/2016/China (14) | ng/ml | 972,46 | 648,36 | 15 | 2183,33 | 615,26 | 9 |
| Garnacho-Montero/2014/Spain (5) | GE/ml | 5490 | 3830 | 76 | 4442 | 5950 | 41 |
| Duplessis/2018/USA (12) | ng/ml | 3900 | 4300 | 190 | 3900 | 1400 | 13 |
| Rannikko/2018/Finland (2) | ng/ml | 1400 | 349,5 | 425 | 2150 | 1080 | 44 |
| Dwivedi/2012/Canada (1) | ng/ml | 1160 | 130 | 46 | 4650 | 480 | 34 |
| Avriel/2014/Israel (11) | ng/ml | 1709 | 1329 | 73 | 4762 | 5294 | 35 |
| Rhodes/2006/UK (7) | ng/ml | 324 | 213,4 | 39 | 1336,75 | 1401 | 13 |
| Jing/2022/China (15) | ng/ml | 206,4181 | 355 | 20 | 1173,58 | 1823 | 10 |
| Lee/2021/Republic of Korea (45) | ng/ml | 2,42 | 0,62 | 9 | 3,12 | 0,47 | 11 |
| Sauer/2018/Germany (26) | ng/ml | 454,35 | 238,9 | 39 | 450,9 | 264 | 12 |
| Kung/2012/Taiwan (28) | ng/ml | 675 | 904 | 56 | 2855 | 2292 | 11 |
| Gao/2019/China(29) | ng/ml | 241,17 | 96,14 | 4 | 582,36 | 160,05 | 11 |

**Supplemental Table 7.** Comparison of prognostic mean and standard deviation (SD) between septic patients and severe septic patients.

| **Study** | **Unit** | **Sepsis Mean** | **Sepsis SD** | **Sepsis sample size** | **Severe sepsis Mean** | **Severe sepsis SD** | **Severe sepsis sample size** |
| --- | --- | --- | --- | --- | --- | --- | --- |
| Moreira/2010/Spain (9) | GE/ml | 25840,38 | 19351,19 | 28 | 53438 | 34876,16 | 26 |
| Duplessis/2018/USA (12) | ng/ml | 3890 | 4236 | 131 | 4800 | 5800 | 35 |
| Patel/2018/UK (24) | ng/ml | 1396,6 | 300 | 39 | 1831 | 619 | 60 |
| Huang/2018/China (8) | ng/ml | 1389,3 | 322,1 | 50 | 3318,3 | 2317,4 | 55 |

**Supplemental Table 8.** Quality assessment of the included studies using the QUADAS-2 tool.

These 28 studies of low risk of bias employed a study cohort design, which effectively avoided unwarranted exclusions. In contrast, two studies remained unclear regarding their risk of bias due to partial lack of information in patient selection (4, 46), while five studies demonstrated a high risk of bias owing to several factors, including inappropriate patient exclusions (47), total lack of information (48, 49), nonconsecutive patient enrollment (1), and retrospective recruitment method (50). The measurement of cfDNA was unbiased in the index test domain because it is an objective measure unaffected by the tester's knowledge of the patient's clinical status. The reference standard for diagnosing sepsis varied among the studies depending on the timeline of the research, in accordance with the prevailing sepsis guidelines (Sepsis-1, Sepsis-2, Sepsis-3). This observation aligns with the unbiased nature of the included studies. With the exception of one study that did not specify the timing of sample collection (16). The remaining studies were considered to have a low risk of bias in the Flow and Timing domain. This is because there was a reliable estimation of the time interval between clinical diagnosis and biomarker measurement.

**Supplemental Table 9.** Quality assessment of the included studies using the QUAPAS tool.

In the domain of participant selection, two studies demonstrated a low risk of bias, one study remained unclear due to inadequate information (11), and one study was considered to be high biased due to inappropriate patient exclusions (12). The remaining domains of index test, outcome assessment, flow/timing, and analysis were deemed to be unbiased as all criteria were satisfactorily met.

**References**

1. Dwivedi DJ, Toltl LJ, Swystun LL, et al: Prognostic utility and characterization of cell-free DNA in patients with severe sepsis. *Crit Care* 2012; 16(4):R151

2. Rannikko J, Seiskari T, Huttunen R, et al: Plasma cell-free DNA and qSOFA score predict 7-day mortality in 481 emergency department bacteraemia patients. *J Intern Med* 2018; 284(4):418-426

3. Maruchi Y, Tsuda M, Mori H, et al: Plasma myeloperoxidase-conjugated DNA level predicts outcomes and organ dysfunction in patients with septic shock. *Crit Care* 2018; 22(1):176

4. Hashiba M, Huq A, Tomino A, et al: Neutrophil extracellular traps in patients with sepsis. *J Surg Res* 2015; 194(1):248-254

5. Garnacho-Montero J, Huici-Moreno MJ, Gutierrez-Pizarraya A, et al: Prognostic and diagnostic value of eosinopenia, C-reactive protein, procalcitonin, and circulating cell-free DNA in critically ill patients admitted with suspicion of sepsis. *Crit Care* 2014; 18(3):R116

6. MeÇO BC, ÜNal MN, Oral M, et al: Can plasma-free DNA concentration be a diagnostic tool in critically ill septic patients? *Turkish Journal of Medical Sciences* 2013; 43(1):150 - 155

7. Rhodes A, Wort SJ, Thomas H, et al: Plasma DNA concentration as a predictor of mortality and sepsis in critically ill patients. *Crit Care* 2006; 10(2):R60

8. Huang T, Yang Z, Chen S, Chen J: [Predictive value of plasma cell-free DNA for prognosis of sepsis]. *Zhonghua Wei Zhong Bing Ji Jiu Yi Xue* 2018; 30(10):925-928

9. Moreira VG, Prieto B, Rodriguez JS, Alvarez FV: Usefulness of cell-free plasma DNA, procalcitonin and C-reactive protein as markers of infection in febrile patients. *Ann Clin Biochem* 2010; 47(Pt 3):253-258

10. Schneck E, Samara O, Koch C, et al: Plasma DNA and RNA differentially impact coagulation during abdominal sepsis-an explorative study. *J Surg Res* 2017; 210:231-243

11. Avriel A, Paryente Wiessman M, Almog Y, et al: Admission cell free DNA levels predict 28-day mortality in patients with severe sepsis in intensive care. *PLoS One* 2014; 9(6):e100514

12. Duplessis C, Gregory M, Frey K, et al: Evaluating the discriminating capacity of cell death (apoptotic) biomarkers in sepsis. *J Intensive Care* 2018; 6(1):72

13. Clementi A, Virzi GM, Brocca A, et al: The Role of Cell-Free Plasma DNA in Critically Ill Patients with Sepsis. *Blood Purif* 2016; 41(1-3):34-40

14. Hou YQ, Liang DY, Lou XL, et al: Branched DNA-based Alu quantitative assay for cell-free plasma DNA levels in patients with sepsis or systemic inflammatory response syndrome. *J Crit Care* 2016; 31(1):90-95

15. Jing Q, Leung CHC, Wu AR: Cell-Free DNA as Biomarker for Sepsis by Integration of Microbial and Host Information. *Clin Chem* 2022; 68(9):1184-1195

16. Pisarev VM, Chumachenko AG, Filev AD, et al: Combination of DNA Molecular Biomarkers in the Prediction of Critical Illness Outcome. *General Reanimatology* 2019; 15(3):31-47

17. Tanaka S, Diallo D, Delbosc S, et al: High-density lipoprotein (HDL) particle size and concentration changes in septic shock patients. *Ann Intensive Care* 2019; 9(1):68

18. Dawulieti J, Sun M, Zhao Y, et al: Treatment of severe sepsis with nanoparticulate cell-free DNA scavengers. *Sci Adv* 2020; 6(22):eaay7148

19. Grumaz S, Grumaz C, Vainshtein Y, et al: Enhanced Performance of Next-Generation Sequencing Diagnostics Compared With Standard of Care Microbiological Diagnostics in Patients Suffering From Septic Shock. *Crit Care Med* 2019; 47(5):e394-e402

20. Cani E, Dwivedi DJ, Liaw KL, et al: Immunothrombosis Biomarkers for Distinguishing Coronavirus Disease 2019 Patients From Noncoronavirus Disease Septic Patients With Pneumonia and for Predicting ICU Mortality. *Crit Care Explor* 2021; 3(12):e0588

21. Lee YY, Park HH, Park W, et al: Long-acting nanoparticulate DNase-1 for effective suppression of SARS-CoV-2-mediated neutrophil activities and cytokine storm. *Biomaterials* 2021; 267:120389

22. Kumar S, Gupta E, Kaushik S, et al: Quantification of NETs formation in neutrophil and its correlation with the severity of sepsis and organ dysfunction. *Clin Chim Acta* 2019; 495:606-610

23. Pan B, Li Y, Liu Y, et al: Circulating CitH3 Is a Reliable Diagnostic and Prognostic Biomarker of Septic Patients in Acute Pancreatitis. *Front Immunol* 2021; 12:766391

24. Patel JM, Sapey E, Parekh D, et al: Sepsis Induces a Dysregulated Neutrophil Phenotype That Is Associated with Increased Mortality. *Mediators Inflamm* 2018; 2018:4065362

25. Yang S, Qi H, Kan K, et al: Neutrophil Extracellular Traps Promote Hypercoagulability in Patients With Sepsis. *Shock* 2017; 47(2):132-139

26. Sauer M, Haubner C, Richter G, et al: Impaired Cell Viability and Functionality of Hepatocytes After Incubation With Septic Plasma-Results of a Second Prospective Biosensor Study. *Front Immunol* 2018; 9:1448

27. Czaikoski PG, Mota JM, Nascimento DC, et al: Neutrophil Extracellular Traps Induce Organ Damage during Experimental and Clinical Sepsis. *PLoS One* 2016; 11(2):e0148142

28. Kung CT, Hsiao SY, Tsai TC, et al: Plasma nuclear and mitochondrial DNA levels as predictors of outcome in severe sepsis patients in the emergency room. *J Transl Med* 2012; 10(1):130

29. Gao F, Hui J, Yang L, et al: [Changes in neutrophil function in septic liver injury and its effect on prognosis: a prospective observational study]. *Zhonghua Wei Zhong Bing Ji Jiu Yi Xue* 2019; 31(11):1324-1329

30. Forsblom E, Aittoniemi J, Ruotsalainen E, et al: High cell-free DNA predicts fatal outcome among Staphylococcus aureus bacteraemia patients with intensive care unit treatment. *PLoS One* 2014; 9(2):e87741

31. Huttunen R, Kuparinen T, Jylhava J, et al: Fatal outcome in bacteremia is characterized by high plasma cell free DNA concentration and apoptotic DNA fragmentation: a prospective cohort study. *PLoS One* 2011; 6(7):e21700

32. Urosevic N, Merritt AJ, Inglis TJJ: Plasma cfDNA predictors of established bacteraemic infection. *Access Microbiol* 2022; 4(6):acmi000373

33. Alkhamis T, Ivic D, Wagner J, et al: Postoperative immunosuppression markers and the occurrence of sepsis in patients with benign and malignant disease. *Wien Klin Wochenschr* 2014; 126(23-24):774-784

34. Jackson Chornenki NL, Coke R, Kwong AC, et al: Comparison of the source and prognostic utility of cfDNA in trauma and sepsis. *Intensive Care Med Exp* 2019; 7(1):29

35. Lehmann-Werman R, Magenheim J, Moss J, et al: Monitoring liver damage using hepatocyte-specific methylation markers in cell-free circulating DNA. *JCI Insight* 2018; 3(12)

36. Abdelaal AA, Elghobary HAF, Ibrahiem SK, Sleem HM: Cell free DNA concentration and serum leptin level as predictors of mortality in a sample of septic Egyptian children. *J Crit Care* 2018; 44:124-127

37. Purhonen AK, Juutilainen A, Vanska M, et al: Human plasma cell-free DNA as a predictor of infectious complications of neutropenic fever in hematological patients. *Infect Dis (Lond)* 2015; 47(4):255-259

38. Saukkonen K, Lakkisto P, Pettila V, et al: Cell-free plasma DNA as a predictor of outcome in severe sepsis and septic shock. *Clin Chem* 2008; 54(6):1000-1007

39. Logters T, Paunel-Gorgulu A, Zilkens C, et al: Diagnostic accuracy of neutrophil-derived circulating free DNA (cf-DNA/NETs) for septic arthritis. *J Orthop Res* 2009; 27(11):1401-1407

40. Hampson P, Dinsdale RJ, Wearn CM, et al: Neutrophil Dysfunction, Immature Granulocytes, and Cell-free DNA are Early Biomarkers of Sepsis in Burn-injured Patients: A Prospective Observational Cohort Study. *Ann Surg* 2017; 265(6):1241-1249

41. Martins GA, Kawamura MT, Carvalho Mda G: Detection of DNA in the plasma of septic patients. *Ann N Y Acad Sci* 2000; 906(1):134-140

42. Kumar R, Katare PB, Lentz SR, et al: Thrombotic potential during pediatric acute lymphoblastic leukemia induction: Role of cell-free DNA. *Res Pract Thromb Haemost* 2021; 5(5):e12557

43. Lenz M, Maiberger T, Armbrust L, et al: cfDNA and DNases: New Biomarkers of Sepsis in Preterm Neonates&mdash;A Pilot Study. *Cells* 2022; 11(2):192

44. Nguyen DN, Stensballe A, Lai JC, et al: Elevated levels of circulating cell-free DNA and neutrophil proteins are associated with neonatal sepsis and necrotizing enterocolitis in immature mice, pigs and infants. *Innate Immun* 2017; 23(6):524-536

45. Li Y, Guo J, Yang H, et al: Comparison of culture-negative and culture-positive sepsis or septic shock: a systematic review and meta-analysis. *Crit Care* 2021; 25(1):167

46. Tanaka S, Diallo D, Delbosc S, et al: High-density lipoprotein (HDL) particle size and concentration changes in septic shock patients. *Annals of Intensive Care* 2019; 9(1):68

47. Cani E, Dwivedi DJ, Liaw K-L, et al: Immunothrombosis Biomarkers for Distinguishing Coronavirus Disease 2019 Patients From Noncoronavirus Disease Septic Patients With Pneumonia and for Predicting ICU Mortality. *Critical Care Explorations* 2021; 3(12):e0588

48. Czaikoski PG, Mota JMSC, Nascimento DC, et al: Neutrophil Extracellular Traps Induce Organ Damage during Experimental and Clinical Sepsis. *PLOS ONE* 2016; 11(2):e0148142

49. Lee YY, Park HH, Park W, et al: Long-acting nanoparticulate DNase-1 for effective suppression of SARS-CoV-2-mediated neutrophil activities and cytokine storm. *Biomaterials* 2021; 267:120389

50. Nguyen DN, Stensballe A, Lai JC, et al: Elevated levels of circulating cell-free DNA and neutrophil proteins are associated with neonatal sepsis and necrotizing enterocolitis in immature mice, pigs and infants. *Innate Immunity* 2017; 23(6):524-536

1. 26 Patients among them had septic shock. [↑](#footnote-ref-1)
2. 110 febrile patients were recruited, only 54 of them were septic. [↑](#footnote-ref-2)
3. Median ICU stay. [↑](#footnote-ref-3)
4. Median hospital stay [↑](#footnote-ref-4)
5. Outcomes were monitored over a period of one year; however, extraction was restricted to the 30-day mortality. [↑](#footnote-ref-5)
6. The median length of hospital stay. [↑](#footnote-ref-6)
7. 21 septic patients without liver injury, 15 septic patients with liver injury. [↑](#footnote-ref-7)
8. Mortality cases were observed only among patients with septic hepatic injury. [↑](#footnote-ref-8)
9. Early onset neonatal sepsis [↑](#footnote-ref-9)
10. Late onset neonatal sepsis [↑](#footnote-ref-10)
11. Pediatric logistic organ dysfunction [↑](#footnote-ref-11)
12. The entire sample consisted of 100 patients diagnosed with Acute Myeloid Leukemia (AML) who experienced neutropenic fever. [↑](#footnote-ref-12)
13. Death due to septic shock [↑](#footnote-ref-13)
14. Different control Group [↑](#footnote-ref-14)
